# Supplementary material for: Transient Effect of Infant Formula Supplementation on the Intestinal Microbiota
Source: Nutrients. 2021 Mar 1;13(3):807. doi: 10.3390/nu13030807 (PMC7998963; doi:10.3390/nu13030807)
Supplement: Supplementary file 1 [file nutrients-13-00807-s001.pdf]

Supplementary Table S1. The average representation of surveyed immune cells at each time points in supplemented and unsupplemented infants.

Time Point	Birth		1 Month		3 Month		6 Month	
**Immunophenotype**	**Unsupp.**	**Supp.**	**Unsupp.**	**Supp.**	**Unsupp.**	**Supp.**	**Unsupp.**	**Supp.**
$CD20^+$ B cells (% Lymphocytes)	4.236	5.107	7.422	6.563	12.160	7.702	6.233	7.951
$CD20^+$ $CD80^+$ B cells (% B cells)	1.168	1.879	1.257	1.435	6.488	1.674	3.370	4.126
$CD4^+$ T cells (% Lymphocytes)	65.000	56.544	68.255	66.438	55.633	69.480	67.318	58.543
$CD4^+$ HLA-DR^+ T cells (% $CD4^+$ T cells)	0.174	0.238	0.734	0.399	0.808	0.526	0.738	0.983
$CD25^+$ $CD127^{low}$ T_{reg} cells (% $CD4^+$ T cells)	4.280	3.798	5.804	4.896	17.293	5.318	5.076	5.454
$CD4^+$ T_{EM} cells (% $CD4^+$ T cells)	1.379	2.028	2.825	2.538	3.632	2.416	2.555	3.041
$CD4^+$ HLA-DR^+ T_{EM} cells (%$CD4^+$ T_{EM} cells)	2.561	4.816	9.391	5.043	12.010	8.258	14.256	15.800
$CD4^+$ T_{MEM} cells (% $CD4^+$ T cells)	1.441	2.687	2.532	2.720	4.082	3.026	2.658	3.271
$CD4^+$ HLA-DR^+ T_{MEM} cells (% $CD4^+$ T_{MEM} cells)	5.985	7.590	10.784	6.874	13.855	9.960	14.720	17.370
$CD8^+$ T cells (% Lymphocytes)	21.175	26.044	24.464	28.075	23.917	21.020	22.911	28.943
$CD8^+$ HLA-DR^+ T cells (% $CD8^+$ T cells)	0.825	1.940	3.315	0.655	3.385	1.606	2.542	1.164
$CD8^+$ T_{EM} cells (% $CD8^+$ T cells)	4.085	6.267	10.331	6.056	14.885	8.118	13.435	9.807
$CD8^+$ HLA-DR^+ T_{EM} cells (% $CD8^+$ T_{EM} cells)	1.974	7.623	11.135	3.158	11.488	9.872	11.034	5.550
$CD8^+$ T_{MEM} cells (% $CD8^+$ T cells)	7.638	11.018	12.752	18.826	8.653	9.688	6.937	3.757
$CD8^+$ HLA-DR^+ T_{MEM} cells (% $CD8^+$ T_{MEM} cells)	2.308	7.876	10.895	2.544	17.682	7.214	14.895	13.213
Total Monocytes (% $CD20^-$ lymphocytes)	30.311	38.364	19.899	24.609	47.755	30.198	28.096	33.311
Total $CD80^+$ Monocytes (% Total monocytes)	3.608	0.944	1.464	4.296	3.702	1.564	3.897	3.884
$CD14^+$ $CD16^-$ Monocytes (% Total monocytes)	10.040	20.159	2.600	3.858	6.267	3.978	2.065	0.814
$CD14^+$ $CD16^-$ $CD80^+$ Monocytes (%$CD14^+$ $CD16^-$ Monocytes)	1.093	0	0.458	0.838	0	0.156	1.606	0.621
$CD14^-$ $CD16^+$ Monocytes (% Total monocytes)	15.891	15.877	16.849	20.063	37.683	23.654	25.832	32.200
$CD14^-$ $CD16^+$ $CD80^+$ Monocytes (% $CD14^-$ $CD16^+$ Monocytes)	1.329	0.944	1.006	1.371	3.702	0.726	2.291	3.263
$CD14^+$ $CD16^+$ Monocytes (% Total monocytes)	4.380	2.329	0.450	0.689	3.805	2.566	0.199	0.297
$CD14^+$ $CD16^+$ $CD80^+$ Monocytes (%$CD14^+$ $CD16^+$ Monocytes)	1.186	0	0	2.088	0	0.682	0	0

Unsupp.: unsupplemented; Supp.: supplemented.
